# The impact of armed conflict on cancer among civilian populations in low- and middle-income countries: a systematic review

**DOI:** 10.3332/ecancer.2020.1039

**Published:** 2020-05-08

**Authors:** Mohammed Jawad, Christopher Millett, Richard Sullivan, Fadel Alturki, Bayard Roberts, Eszter P Vamos

**Affiliations:** 1 Public Health Policy Evaluation Unit, Imperial College London, Hammersmith, London W6 8RP, UK; 2 Institute of Cancer Policy, Cancer Epidemiology, Population and Global Health, King's College London and Guy's & St Thomas' NHS Trust, London, UK; 3 Faculty of Medicine, American University of Beirut, Lebanon; 4 Department of Health Services Research and Policy, London School of Hygiene and Tropical Medicine, London WC1H 9SH, UK

**Keywords:** cancer, conflict, war, systematic review, low-income countries, middle-income countries

## Abstract

**Background:**

Armed conflicts are increasingly impacting countries with a high burden of cancer. The aim of this study is to systematically review the literature on the impact of armed conflict on cancer in low- and middle-income countries (LMICs).

**Methods:**

In November 2019, we searched five medical databases (Embase, Medline, Global Health, PsychINFO and the Web of Science) without date, language or study design restrictions. We included studies assessing the association between armed conflict and any cancer among civilian populations in LMICs. We systematically re-analysed the data from original studies and assessed quality using the Newcastle-Ottawa Scale. Data were analysed descriptively by cancer site.

**Results:**

Of 1,543 citations screened, we included 20 studies assessing 8 armed conflicts and 13 site-specific cancers (total study population: 70,172). Two-thirds of the studies were of low methodological quality (score <5) and their findings were often conflicting. However, among outcomes assessed by three or more studies, we found some evidence that armed conflict was associated with increases in the incidence and mortality of non-specific cancers, breast cancer and cervical cancer. Single studies reported a positive association between armed conflict and the incidence of stomach and testicular cancers, some as early as 3 years after the onset of conflict. Some studies reported a post-conflict impact on time to diagnosis.

**Conclusion:**

Our findings support the need for more rigorous longitudinal and cohort studies of populations in and immediately post-conflict to inform the development of basic packages of cancer services, and post-conflict cancer control planning and development.

## Introduction

Cancer caused 8.7 million deaths globally in 2015, making it the second leading cause of death after cardiovascular disease [1]. Although this figure is likely to be an underestimate [2], the burden of cancer is increasing in low- and middle-income countries (LMICs), where 80% of the world’s population live [3] and where about two-thirds of all cancer deaths occur [4]. This is due to increasing life expectancy coupled with changing patterns of behavioural risk factors associated with higher non-communicable disease risk, such as tobacco and alcohol use, obesity, physical inactivity and an unhealthy diet [5]. Occupational, environmental and dietary exposure to carcinogens also account for substantial numbers of cancer deaths [2]. Calls for better cancer prevention and early diagnosis and better treatment all form part of Target 3.4 of the Sustainable Development Goals (SDGs), which aims for a one-third reduction in premature mortality from non-communicable diseases by 2030 [6].

Efforts to meet SDG Target 3.4, and indeed other SDGs, are likely to be hampered by the presence of armed conflict. In 2018, there were 52 armed conflicts where at least one party was a government of state, and a record 82 active civil wars [7]. Although the number of armed conflicts has been increasing, the number of deaths occurring in armed conflicts has been markedly decreasing. Armed conflicts may increase cancer incidence, complications and mortality in the short term by disrupting patients seeking care and the delivery of all aspects of oncological care [9, 10]. Additional impacts on cancer services may result from sudden demographic shifts associated with armed conflict and forced migration (internally displaced persons or refugees). This may increase late diagnoses for potentially curable site-specific cancers, abandonment of treatment or sub-optimal treatment, all of which increase the burden of cancer on patients and health services.

Longer-term impacts of armed conflict on cancer incidence may also be a result of the toxic contamination of the environment. Examples include the Vietnam War, where 10% of south Vietnam was sprayed with the carcinogenic Agent Orange [11] and the Second World War where atomic bombs were dropped on the Japanese cities of Hiroshima and Nagasaki [12]. Furthermore, stress experienced during armed conflict may encourage unhealthy behaviours that increase the risk of cancer, such as tobacco and alcohol use [16–18]. Finally, mass population displacement increases the risk of communicable disease transmission, which can increase the infectious causes of cancer, such as human papillomavirus and chlamydia trachomatis (cervical cancer), Epstein–Barr virus (nasopharyngeal cancer and lymphomas), hepatitis B and C (liver cancer, non-Hodgkin lymphoma) and others.

The greater number and increasingly protracted nature of conflict globally warrants a better understanding of its relationship to cancer care and cancer mortality. Understanding the relationship between armed conflict and cancer is important as more conflicts occur in demographically and epidemiologically transitioned societies. It remains unclear which short- or long-term approaches are most important in mediating the impact of armed conflict on cancer burden, and whether any of these factors are feasibly modifiable during an active conflict or in the post-conflict setting. This study aimed to review the literature for the impact of armed conflict on cancer, in particular its incidence and mortality among civilians in LMICs.

## Methods

This systematic review is registered on Prospero (ID: CRD42017065722) and follows the PRISMA reporting standards [20]. Our research questions is: ‘What is the association between armed conflict and cancer for civilians in LMICs, compared to civilians with less or no exposure to armed conflict?’

### Search strategy and selection criteria

We searched five electronic databases (Embase, Medline, Global Health, PsychINFO and the Web of Science) in November 2019 without language or date restrictions, using synonyms for armed conflict, cancer and LMICs. The full search strategy can be found in Table S1. We also hand-searched citation lists of included studies to identify additionally relevant articles. In line with previous reviews, we did not search the grey literature given the limited information available [21].

The inclusion criteria comprised civilian populations (including children, internally displaced persons, and refugees) in LMICs exposed to author-defined armed conflict with a diagnosis of any type of cancer. We did not exclude studies by design but a component of comparison to a non- or less-conflict exposed group was required for eligibility. In the case of ecological studies collecting serial data points over time (e.g., hospital admission data pre-, during- and post-conflict), we excluded studies whose first post-conflict data point was greater than 3 years after the end of the conflict.

We excluded studies reporting on military veterans, combatants and studies from high-income countries (including where refugees had migrated to high-income countries). We also excluded studies whose exposure was weapons (often, nuclear) testing rather than armed conflict. Studies that mentioned armed conflict but did not attempt to measure it were further excluded.

### Data analysis

Two reviewers performed all citation screening and data abstraction in duplicate and independently using pilot-tested forms. Disagreements were resolved by discussion, and when needed with the help of a third reviewer. We retrieved full texts of citations considered eligible by at least one reviewer. Data extracted from eligible studies included study provenance (funding source, ethics approval and conflicts of interest), study features (design, timing, conflict, country and level of jurisdiction), population (sample size, mean age/age range and percentage of males) and results (outcome measure definition, outcome measure effect size and precision). We calculated the maximum number of years from the onset or end of conflict to the time of data collection, to give an indication of the length of armed conflict exposure. We used the Newcastle-Ottawa Scale (NOS) [22–24] to assess the quality of each study. The NOS has been recommended for use for non-randomised studies by the Cochrane Collaboration [25]. Although the NOS has no established threshold of quality, in line with previous reviews [26, 27], we defined studies as low quality (score <5), moderate quality (score 5–6) and high quality (score >6) to simplify the main analysis. Quality scores by NOS domains (selection, comparability and outcome) for each study are detailed in Table S2.

Meta-analysis was not feasible given the degree of between-study heterogeneity in design, armed conflict, population and outcome. We, therefore, analysed data descriptively. To standardise our analytical approach and to reduce bias, we systematically re-analysed reported data and presented a single effect estimate per outcome per study where possible. This included constructing 95% confidence intervals around all effect estimates and considering confidence intervals that did not overlap as statistically significant at an alpha level of 0.05. This also meant we combined outcomes stratified by population subgroups (e.g., by age and sex), and used the overall outcome in our analysis. We did not reanalyse data already presented as odds ratios, beta-coefficients or hazard ratios. Where data were available pre- during- and post-conflict, we used a single estimate for the differences between the pre- versus during-conflict data for each study. Furthermore, an analysis of post-conflict data was undertaken separately to understand better changes in trends throughout the conflict cycle. Each outcome from each study was assigned a qualitative effect direction (increase, decrease or no change) following exposure to armed conflict based on the statistical significance of effects. We stratified our analysis by cancer incidence and mortality, and outcomes with greater than three studies were described in more detail and displayed graphically using Harvest plots. Harvest plots take aspects of a forest plot to display data on a matrix of effect direction weighted by several variables [28]. Finally, we visually assessed publication bias by constructing an adapted funnel plot, using the sample size and the qualitative effect direction in place of the standard error and effect size, respectively.

## Results

### Study characteristics

**Figure 1.**
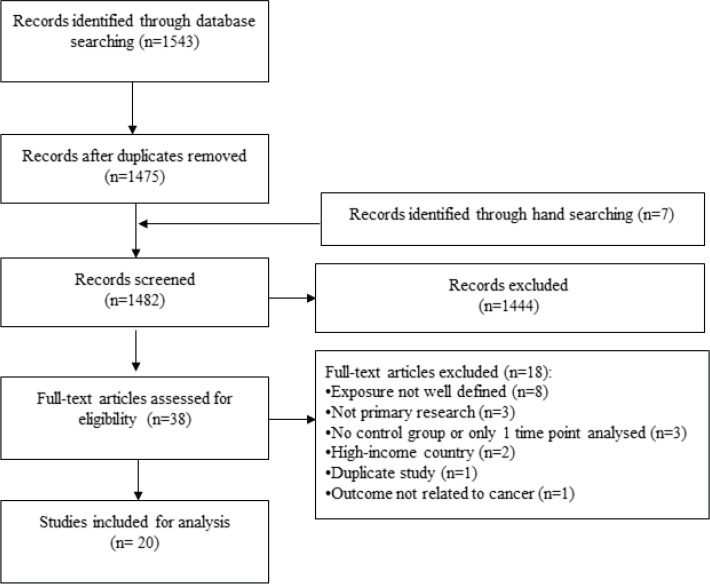
Study flow.

Of 1,543 records identified through database searching, 38 were potentially eligible and 20 were included in the final analysis (Figure 1). The total study population was 70,172. Three-quarters of studies used an ecological design (75.0%) and over one-third analysed the Croatian War of Independence (1991–1995) (35.0%). Over half were conducted in cities (55.0%) and 70.0% utilised hospital-derived data. The average follow-up time was 16.8 years (range 3–64 years) and study quality was mostly rated as low (65.0%). Only four outcomes were assessed by three or more studies: the incidence of any, breast and cervical cancer, and mortality from any cancer.

### Incidence of any cancer

**Figure 2.**
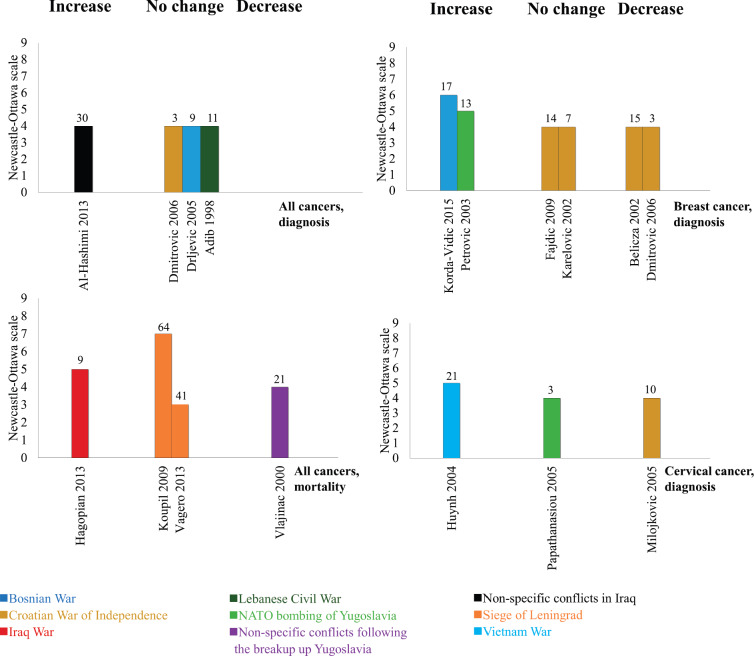
The impact of armed conflict on cancer incidence and mortality. Interpretation: Height refers to study quality, colour refers to armed conflict, number refers to length of follow-up between conflict exposure and outcome, bars grouped as showing either an increase, decrease, or no change following exposure to armed conflict.

Four studies, all low quality and ecological, assessed the incidence of any type of cancer (Figure 2, top left panel). One subnational cancer registry study analysed non-specific conflicts in Iraq over 30 years and showed an increase in the incidence rate ratio of cancers throughout the conflict and into the post-conflict period [29]. It did not compare incidence rate ratios in similar countries not at war during this period of time. Two hospital-based studies from the Balkans showed no change in cancer incidence during the conflict compared to the pre-conflict baseline [30, 31]. Another cancer registry study assessed the Lebanese Civil War and showed no change in cancer incidence during the conflict period (1983–1991, mean 786 cases/year) compared to the post-conflict period (1992 to 1994, mean 802.3 cases/year) [32].

### Mortality from any cancer

Four studies assessed mortality from any cancer (Figure 2, bottom left panel). One moderate-to-high quality study assessed the 2003 US-led invasion of Iraq and reported an average 50% increase in the number of households reporting cancer deaths from the pre-conflict period (mean 9.9 cases/year in 2001–2002) to the conflict period (mean 14.8 cases/year in 2003–2010) [33]. We calculated this difference to be statistically significant (4.9 cases/year, 95% CI 0.4–9.4). Two survivor cohort studies from the Siege of Leningrad (1941–1944) reported no change in cancer mortality 41 to 64 years after the siege although both adjusted hazard ratios showed positive effect estimates (1.12 (95% CI 0.95 -1.31) and 1.11 (95% CI 0.97 -1.27)) [34, 35]. One modelling study (1973 to 1994) used data from the Federal Institute of Statistics to assess the impact of the breakup of Yugoslavia, and found that cancer mortality decreased during periods of war and sanctions [36].

### Breast cancer incidence

Six studies, all assessing wars in the Balkans during the 1990s, reported on breast cancer incidence (Figure 2, top right panel). Both moderate-to-high quality studies showed an increase in breast cancer incidence [37, 38]. One of these was ecological in design, monitored trends 13 years before the 1999 NATO bombing of Yugoslavia, and reported an increase from an average of 67.2 cases/year before the conflict to 80.2 cases/year during the conflict [38]. We calculated this difference to be statistically significant (13.0 cases/year, 95% CI 4.1–21.9). The other study used a case-control design and reported increased odds of breast cancer among those with greater exposure to war-related events in Bosnia (pooled odds across all events: 1.55, 95% CI 1.37–1.73) [37]. The remaining four studies, all low quality and ecological in design, showed no change [39, 40] or a decrease [31, 41] in breast cancer incidence. The study with the shortest follow-up in this review (3 years) was one study that showed a decrease in breast cancer diagnosis during the Croatian War of Independence (32 cases in 2 years) compared to the pre-conflict baseline (86 cases in 2 years) [31]. We considered this decrease statistically significant (−54.0 cases/2 years, 95% CI–75.3 to −32.7).

### Cervical cancer incidence

Three studies assessed cervical cancer incidence (Figure 2, bottom right panel). One moderate-to-high quality case-control study of the Vietnam War showed that women with a husband in the army had higher odds of cervical cancer compared to those without (adjusted odds ratio (AOR) 1.32, 95% CI: 1.00–1.75) [42]. One low-quality ecological study in Greece assessed over 35,000 smear tests from hospitals with different proximity to the Yugoslav border, but showed no difference in either cervical cancer or cervical intraepithelial neoplasia incidence between the sites following the NATO bombing of Yugoslavia in 1999 [43]. Another low-quality hospital-based ecological study found a decrease in cervical cancer incidence, from 214 cases in 6 years before the Croatian war, to 142 in 6 years of the war [44]. We found this to be a statistically significant decrease (−72.0, 95% CI: −109.0 to −35.0).

### Other cancers

Eight studies examined other site-specific cancers, but they were too few to display graphically and describe collectively. One hospital-based study from Croatia reported a rise in the incidence of malignant stomach and testicular cancers when comparing 2 years of conflict to 2 years prior [31]. Other studies of various study design and quality found no association between armed conflict and mortality from breast cancer [34, 35], colon cancer [34], lung cancer [34, 35] and stomach cancer [34], nor the incidence of corpus cancer [44], haematological cancers [45], lung cancer [31], pancreatic cancer [31] and prostate cancer [34]. One study reported a decrease in the incidence of colon cancer [31]. Finally, four studies reported mixed evidence for changes in the incidence of intracranial [46, 47], oropharyngeal [48] and ovarian [31, 44] cancers.

### Post-conflict trends

All seven studies that assessed the conflict cycle (i.e., pre-conflict, conflict and post-conflict) were ecological, hospital-based studies analysing either the Croatian or Bosnian wars of the 1990s [30, 39, 41, 44–47]. The three studies that reported no change between the times before and during the conflict then showed an increase in incidence in the post-conflict period [30, 39, 44]. The one study that reported an increase in incidence between the pre- and during-conflict periods found that this increase was sustained into the post-conflict period [47]). In the three studies that reported a decrease in incidence between the pre- and during-conflict periods found that this either plateaued [41, 46] or returned to pre-conflict levels [44] during the post-conflict period. One ecological study showed mixed findings in the incidence of haematological cancers depending on the type of conflict exposure used (areas affected by depleted uranium, chemical damage or population mixing) and outcome (Hodgkin’s lymphoma, non-Hodgkin’s lymphoma, lymphatic leukaemia and myeloid leukaemia), but generally found either no change or a decrease in incidence through the post-conflict period [45].

### Publication bias

**Figure 3.**
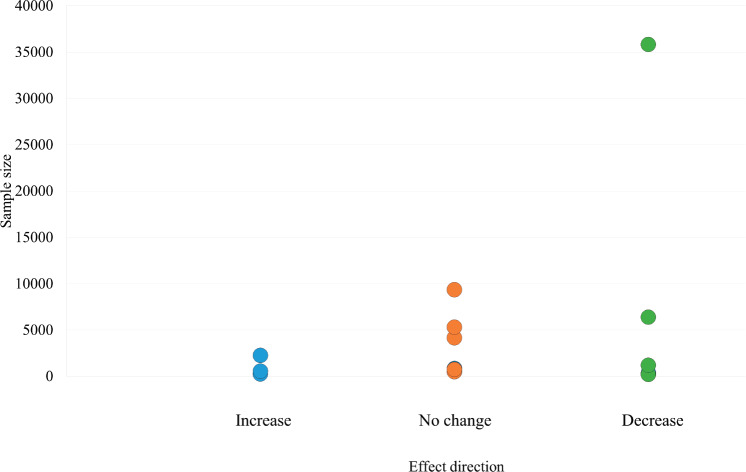
Adapted funnel plot assessing publication bias.

Figure 3 presents an adapted funnel plot to assess publication bias, which includes all 55 outcomes from the 20 included studies. While the absence of actual effect estimates limits interpretation, the plot does not present convincing evidence of asymmetry or the absence of small studies showing no effect, which are indicative of publication bias.

## Discussion

The literature on the impact of armed conflict on cancer incidence and mortality is very sparse, methodologically poor, and often contradictory. This is despite the fact that some have extensive follow-up periods, which averaged 18 years. The main limitations to many studies were their design, namely, ecological, and thus subject to ecological fallacies; nearly all failed to acknowledge this, in addition to failing to account for sudden population demographic changes following forced migration. There was also limited adjustment for confounding variables in risk factor exposure and behaviour changes. The lack of data on factors, which may mediate the impact of armed conflict on cancer, is an additional serious limitation in the extant literature.

The one cancer (breast) that did have several studies showing an increase in incidence following armed conflict did not have, however, sufficient data to advance understanding of plausible aetiological factors. Armed conflict has been shown to change reproductive strategies in populations affected with greater parity and lower maternal age, both of which are protective of breast cancer [49]. Thus it is unclear, whether the increased incidence of breast cancer is real or an artefact.

The factors that affect cancer incidence and mortality in armed conflict are multifactorial and multilevel; these includes changes to risk factor exposure, behavioural changes, delays to presentation, the availability of timely and affordable complex care (depending on the site-specific cancer), the ability to access care, etc. Furthermore, the ability to collect reliable data from registries, hospitals or camps can be substantially hampered during periods of conflict. In some cases, this is because systems are destroyed, data are not collected (too costly or to protect patients identities) or because care data are fragmented across multiple disconnected places of care [50, 51]. Reported data may be inaccurate due to limited diagnostic facilities and available pathologists, so any statistical inference should provide a contextual interrogation to the quality of the data. Reduced case ascertainment featured prominently as a serious lacunae in data collected during the Lebanese Civil War (1975–1991), when the American University of Beirut Medical Center (AUBMC) was the only functioning cancer referral site in the entire county and it was estimated at least two-thirds of the cancer burden during this period went either undiagnosed or unreported [32]. AUBMC and other cancer centres only become accessible after the end of the conflict [32], so any increase in incidence during the post-conflict period may simply reflect a return of the status quo. A similar conclusion was reached in analysing the cancer incidence data collected during the Croatian War of Independence; road blockades across the country and the removal of free care services such as breast cancer check-ups radically reduced health service accessibility [40]. In another analysis of the same conflict, an observed post-conflict increase in cancer incidence was also attributed to the introduction of a new cancer screening programme, better organisation of cancer care services and the introduction of more accurate and up-to-date diagnostic equipment in hospitals [39].

In armed conflict, there is an expected rise in cancer-related mortality due to the loss of skilled personnel, the shift of such personnel into acute care, shortage or failure of key equipment—diagnostic imaging, surgical instruments, radiotherapy and cancer drugs, for example—and the inability of patients to access what care remains due to security or affordability barriers, all factors that led to the rise in cancer mortality during the armed conflict in Serbia in 1999 [38]. Yet it is possible that the same factors that worsen cancer mortality are the same that inhibit the timely and accurate reporting of such mortality, which may explain why many of the studies included in this review reported no change in the incidence or mortality of cancer during or after armed conflict.

Better quality research to study cancer in armed conflict is essential, and our review findings have several research implications. Although resources are often scarce in conflict settings, making use of hospital-based registries or other sources of routinely collected data have excellent potential for robust inquiry. In instances where control groups are not feasible, data could be subject to interrupted time series or difference-in-difference analyses with adjustment for confounders or with age-/sex-standardised rates of cancer incidence. Importantly, researchers should outline the status of screening programmes and other mediators in the relationship between armed conflict and cancer, so that these can be appropriately accounted for in the study design. This will make a more informative contribution to the current literature which is lacking in methodological rigour and often reports crude numbers over time. One notable absence from the literature was studies from humanitarian organisations. Although often unable to collect pre-conflict data, they are in a strong position to assess the degree of conflict exposure among their patients using tools such as the Harvard Trauma Questionnaire [52]. Future research could assess the impact of armed conflict on stage of diagnosis, in addition to inequalities by socioeconomic groups (e.g. age, sex, residence and deprivation). Most studies with very long follow-up times (>30 years) hypothesised that *in utero*, infant or adolescent exposure to armed conflict would have a greater impact on cancer risk to those exposed at older ages [34, 35, 53]. However, the failure to properly control for the many confounders has seriously hampered research to examine the link between toxic contamination of the environment due to armed conflict and long-term health impacts such as cancer.

Our findings also have important policy implications. Despite a number of guidance documents on cancer care in complex emergencies and post disaster, e.g., post typhoon Haiyan issued by WHO [54, 55] the literature is silent on what might constitute basic packages of cancer care, for UN and international NGOs for example and on approaches to post-conflict cancer systems reconstruction, or in supporting host countries absorb and provide care to refugees in both formal and informal (sans papier) settings. Although, it is to be recognised that the latter is intimately linked to post-conflict health systems reconstruction *per se*. More research is needed to urgently inform cancer policies and planning in the context of armed conflicts, particularly now that so many are occurring in high-burden countries with populations that have gone through the demographic and epidemiological transitions.

## Conflicts of interest

The authors have declared no conflicts of interest.

## Funding

This work was supported by the Medical Research Council Doctoral Training Partnership. The Public Health Policy Evaluation Unit, Imperial College London is supported by the NIHR School of Public Health Research. RS is funded through the UK Research and Innovation GCRF RESEARCH FOR HEALTH IN CONFLICT (R4HC-MENA); developing capability, partnerships and research in the Middle and Near East (MENA) ES/P010962/1. The funders had no role in the design, analysis or writing of this manuscript, nor the decision to submit for publication. The corresponding author (MJ) has full access to all the data in the study and had final responsibility for the decision to submit for publication.

## Figures and Tables

**Table S1.** Search strategy: Medline, Embase, PsychInfo, Global Health.

1	exp Neoplasms/	7676601	Advanced
2	cancer*.tw.	3996712	Advanced
3	neoplas*.tw.	776165	Advanced
4	tumo*.tw.	3765025	Advanced
5	carcinoma*.tw.	1485187	Advanced
6	hodgkin*.tw.	157060	Advanced
7	nonhodgkin*.tw.	526	Advanced
8	adenocarcinoma*.tw.	332994	Advanced
9	leukemia*.tw.	499875	Advanced
10	leukaemia*.tw.	104081	Advanced
11	metastat*.tw.	515966	Advanced
12	sarcoma*.tw.	303922	Advanced
13	teratoma*.tw.	34024	Advanced
14	malignan*.tw.	1308813	Advanced
15	lymphoma*.tw.	407427	Advanced
16	melanoma*.tw.	258240	Advanced
17	myeloma*.tw.	127994	Advanced
18	oncolog*.tw.	364356	Advanced
19	Armed Conflict/	31610	Advanced
20	exp Warfare/	54929	Advanced
21	exp War Exposure/	546	Advanced
22	((armed or zone) adj2 conflict*).tw.	3756	Advanced
23	war.tw.	115433	Advanced
24	wars.tw.	11819	Advanced
25	(“conflict affected” adj3 (population* or person* or communit*)).mp. [mp=ti, ab, hw, tn, ot, dm, mf, dv, kw, fx, dq, bt, id, cc, nm, kf, px, rx, ui, sy, tc, tm]	280	Advanced
26	wartime.tw.	5286	Advanced
27	warfare.tw.	13281	Advanced
28	or/19–27	187756	Advanced
29	Developing Countries.sh,kf.	86834	Advanced
30	((developing or less* developed or under developed or underdeveloped or middle income or low* income or underserved or under served or deprived or poor*) adj (countr* or nation? or population? or world)).ti,ab.	254072	Advanced
31	(low* adj (gdp or gnp or gross domestic or gross national)).ti,ab.	635	Advanced
32	(low adj3 middle adj3 countr*).ti,ab.	31781	Advanced
33	(lmic or lmics or third world or lami countr*).ti,ab.	16495	Advanced
34	transitional countr*.ti,ab.	497	Advanced
35	Cambodia/	9676	Advanced
36	(cambodia* or Kampuchea).cp,in,jw,mp.	16070	Advanced
37	“Democratic People’s Republic of Korea”/	948	Advanced
38	(north korea* or (democratic people* republic adj2 korea)).cp,in,jw,mp.	2909	Advanced
39	Myanmar/	7472	Advanced
40	(myanmar or burma or burmese).cp,in,jw,mp.	14657	Advanced
41	Fiji/	2699	Advanced
42	fiji*.cp,in,jw,mp.	7124	Advanced
43	Indonesia/	31608	Advanced
44	indonesia*.cp,in,jw,mp.	70992	Advanced
45	Micronesia/	2722	Advanced
46	(Micronesia* or Kiribati).cp,in,jw,mp.	4452	Advanced
47	Laos/	4998	Advanced
48	(laos or (lao adj1 democratic republic) or (lao adj2 people) or marshall island*).cp,in,jw,mp.	8856	Advanced
49	Mongolia/	5352	Advanced
50	mongolia*.cp,in,jw,mp.	33254	Advanced
51	Papua New Guinea/	12436	Advanced
52	Papua New Guinea.cp,in,jw,mp.	18499	Advanced
53	Philippines/	23256	Advanced
54	(Philippines or filipino*).cp,in,jw,mp.	56650	Advanced
55	samoa/ or “independent state of samoa”/	1436	Advanced
56	samoa*.cp,in,jw,mp.	4406	Advanced
57	Melanesia/	6561	Advanced
58	(Solomon Islands or Timor-Leste or Melanesia*).cp,in,jw,mp.	10868	Advanced
59	Tonga/	780	Advanced
60	tonga*.cp,in,jw,mp.	2796	Advanced
61	Vanuatu/	1076	Advanced
62	Vanuatu.cp,in,jw,mp.	1929	Advanced
63	Vietnam/	31470	Advanced
64	Vietnam*.cp,in,jw,mp.	63695	Advanced
65	exp China/	488990	Advanced
66	(china or chinese).cp,in,jw,mp.	4029109	Advanced
67	Malaysia/	43933	Advanced
68	Malaysia*.cp,in,jw,mp.	161810	Advanced
69	Palau/	517	Advanced
70	(Palau or Belau or Pelew).cp,in,jw,mp.	2785	Advanced
71	Thailand/	74175	Advanced
72	(Thailand or thai*).cp,in,jw,mp.	258491	Advanced
73	(tuvalu or ellice islands).cp,in,jw,mp.	252	Advanced
74	Kyrgyzstan/	3071	Advanced
75	(kyrgyzstan or kyrgyz or kirghizia or kirghiz).cp,in,jw,mp.	5329	Advanced
76	Tajikistan/	1997	Advanced
77	(tajikistan or tadzhik or tadzhikistan or tajikistan).cp,in,jw,mp.	3145	Advanced
78	Albania/	3252	Advanced
79	Albania*.cp,in,jw,mp.	7781	Advanced
80	Armenia/	3513	Advanced
81	Armenia*.cp,in,jw,mp.	15700	Advanced
82	“Georgia (Republic)”/	3447	Advanced
83	georgia*.cp,in,jw,mp.	309463	Advanced
84	Yugoslavia/	20384	Advanced
85	(Jugoslavija* or Yugoslavia* or serbo-croat* or macedonia* or sloven* or kosovo).cp,in,jw,mp.	182508	Advanced
86	Moldova/	2093	Advanced
87	Moldova*.cp,in,jw,mp.	7233	Advanced
88	Ukraine/	33163	Advanced
89	Ukrain*.cp,in,jw,mp.	177783	Advanced
90	Uzbekistan/	4970	Advanced
91	Uzbekistan.cp,in,jw,mp.	9683	Advanced
92	Azerbaijan/	3477	Advanced
93	Azerbaijan*.cp,in,jw,mp.	10050	Advanced
94	“Republic of Belarus”/	4521	Advanced
95	(belarus or byelarus or belorussia).cp,in,jw,mp.	18740	Advanced
96	Bosnia-Herzegovina/	5557	Advanced
97	bosnia*.cp,in,jw,mp.	26942	Advanced
98	Bulgaria/	19189	Advanced
99	Bulgaria*.cp,in,jw,mp.	132182	Advanced
100	Kazakhstan/	7280	Advanced
101	(Kazakhstan or kazakh).cp,in,jw,mp.	15369	Advanced
102	Latvia/	4309	Advanced
103	Latvia*.cp,in,jw,mp.	14271	Advanced
104	Lithuania/	7989	Advanced
105	Lithuania*.cp,in,jw,mp.	32645	Advanced
106	“Macedonia (Republic)”/	1499	Advanced
107	Macedonia*.cp,in,jw,mp.	18485	Advanced
108	Montenegro/	1011	Advanced
109	Montenegro.cp,in,jw,mp.	12126	Advanced
110	Romania/	29547	Advanced
111	Romania*.cp,in,jw,mp.	192775	Advanced
112	exp Russia/	121816	Advanced
113	USSR/	100452	Advanced
114	(russia* or ussr or soviet or cccp).cp,in,jw,mp.	1730511	Advanced
115	Serbia/	10350	Advanced
116	serbia*.cp,in,jw,mp.	102530	Advanced
117	Turkey/	62130	Advanced
118	turk*.cp,in,jw,mp. not animal/	704949	Advanced
119	Turkmenistan/	1504	Advanced
120	Haiti/	8175	Advanced
121	Haiti/	8175	Advanced
122	Haiti.cp,in,jw,mp.	11219	Advanced
123	Belize/	1561	Advanced
124	Belize.cp,in,jw,mp.	2633	Advanced
125	Bolivia/	7577	Advanced
126	Bolivia*.cp,in,jw,mp.	14352	Advanced
127	El Salvador/	3218	Advanced
128	El Salvador.cp,in,jw,mp.	6430	Advanced
129	Guatemala/	9325	Advanced
130	Guatemala*.cp,in,jw,mp.	16832	Advanced
131	Guyana/	1884	Advanced
132	Guyana*.cp,in,jw,mp.	4005	Advanced
133	Honduras/	3571	Advanced
134	Hondura*.cp,in,jw,mp.	6496	Advanced
135	Nicaragua/	4511	Advanced
136	Nicaragua.cp,in,jw,mp.	6910	Advanced
137	Paraguay/	2836	Advanced
138	Paraguay.cp,in,jw,mp.	9550	Advanced
139	“Antigua and Barbuda”/	323	Advanced
140	(Antigua or Barbuda).cp,in,jw,mp.	2739	Advanced
141	Argentina/	43847	Advanced
142	Argentin*.cp,in,jw,mp.	318227	Advanced
143	Brazil/	251275	Advanced
144	Brazil*.cp,in,jw,mp.	1132518	Advanced
145	Chile/	33657	Advanced
146	Chile*.cp,in,jw,mp.	163635	Advanced
147	Colombia/	32939	Advanced
148	Colombia*.cp,in,jw,mp.	99092	Advanced
149	Costa Rica/	9961	Advanced
150	Costa Rica*.cp,in,jw,mp.	25767	Advanced
151	Cuba/	36428	Advanced
152	Cuba*.cp,in,jw,mp.	56766	Advanced
153	Dominica/	346	Advanced
154	Dominican Republic/	4480	Advanced
155	Dominica*.cp,in,jw,mp.	12555	Advanced
156	Ecuador/	9999	Advanced
157	Ecuador*.cp,in,jw,mp.	21748	Advanced
158	Grenada/	497	Advanced
159	Grenad*.cp,in,jw,mp.	5679	Advanced
160	Jamaica/	8829	Advanced
161	Jamaica*.cp,in,jw,mp.	32769	Advanced
162	Mexico/	94503	Advanced
163	Mexic*.cp,in,jw,mp.	510292	Advanced
164	exp Panama/	6410	Advanced
165	Peru/	24083	Advanced
166	Peru*.cp,in,jw,mp.	121963	Advanced
167	Saint Lucia/	370	Advanced
168	(St Lucia* or Saint Lucia*).cp,in,jw,mp.	31049	Advanced
169	“Saint Vincent and the Grenadines”/	188	Advanced
170	Grenadines.cp,in,jw,mp.	388	Advanced
171	Suriname/	2500	Advanced
172	Surinam*.cp,in,jw,mp.	5356	Advanced
173	Uruguay/	6062	Advanced
174	Uruguay.cp,in,jw,mp.	33620	Advanced
175	Venezuela/	15615	Advanced
176	Venezuela*.cp,in,jw,mp.	62459	Advanced
177	Djibouti/	765	Advanced
178	Djibouti.cp,in,jw,mp.	1328	Advanced
179	Egypt/	45378	Advanced
180	Egypt*.cp,in,jw,mp.	256076	Advanced
181	Iraq/	14342	Advanced
182	Iraq*.cp,in,jw,mp.	41190	Advanced
183	Morocco/	16925	Advanced
184	Morocc*.cp,in,jw,mp.	53607	Advanced
185	Syria/	4345	Advanced
186	(Syria* or gaza*).cp,in,jw,mp.	46343	Advanced
187	Yemen/	4328	Advanced
188	yemen*.cp,in,jw,mp.	8741	Advanced
189	Algeria/	9423	Advanced
190	Algeria*.cp,in,jw,mp.	28210	Advanced
191	Iran/	103314	Advanced
192	Iran*.cp,in,jw,mp.	430404	Advanced
193	Jordan/	11829	Advanced
194	jordan*.cp,in,jw,mp.	82444	Advanced
195	Lebanon/	10587	Advanced
196	Leban*.cp,in,jw,mp.	81259	Advanced
197	Libya/	3479	Advanced
198	Libya*.cp,in,jw,mp.	9106	Advanced
199	Tunisia/	21063	Advanced
200	Tunisia*.cp,in,jw,mp.	75717	Advanced
201	Afghanistan/	9699	Advanced
202	Afghan*.cp,in,jw,mp.	21898	Advanced
203	Bangladesh/	32929	Advanced
204	Bangladesh*.cp,in,jw,mp.	67526	Advanced
205	Nepal/	22054	Advanced
206	Nepal*.cp,in,jw,mp.	40842	Advanced
207	Bhutan/	1384	Advanced
208	Bhutan*.cp,in,jw,mp.	4424	Advanced
209	exp India/	328701	Advanced
210	india*.cp,in,jw,mp.	2185658	Advanced
211	Pakistan/	51913	Advanced
212	Pakistan*.cp,in,jw,mp.	188131	Advanced
213	Sri Lanka/	17094	Advanced
214	Sri Lanka*.cp,in,jw,mp.	34681	Advanced
215	Indian Ocean Islands/	6825	Advanced
216	Maldiv*.cp,in,jw,mp.	1041	Advanced
217	Benin/	5525	Advanced
218	(Benin or Dahomey).cp,in,jw,mp.	19616	Advanced
219	Burkina Faso/	10413	Advanced
220	(Burkina Faso or Burkina Fasso or Upper Volta).cp,in,jw,mp.	17067	Advanced
221	Burundi/	1927	Advanced
222	Burundi*.cp,in,jw,mp.	2942	Advanced
223	Central African Republic/	2420	Advanced
224	(Central African Republic or Ubangi-Shari or african*).cp,in,jw,mp.	680868	Advanced
225	Chad/	2287	Advanced
226	Chad.cp,in,jw,mp.	11223	Advanced
227	Comoros/	820	Advanced
228	(comoros or comores).cp,in,jw,mp.	1314	Advanced
229	“Democratic Republic of the Congo”/	10599	Advanced
230	(congo* or zaire).cp,in,jw,mp.	54233	Advanced
231	Eritrea/	1024	Advanced
232	Eritrea*.cp,in,jw,mp.	5415	Advanced
233	Ethiopia/	33164	Advanced
234	Ethiopia*.cp,in,jw,mp.	50473	Advanced
235	Gambia/	7090	Advanced
236	Gambia*.cp,in,jw,mp.	27765	Advanced
237	Guinea/	6270	Advanced
238	(Guinea* not (New Guinea or Guinea Pig* or Guinea Fowl)).cp,in,jw,mp.	18108	Advanced
239	Guinea-Bissau/	2564	Advanced
240	(Guinea-Bissau or Portuguese Guinea).cp,in,jw,mp.	3632	Advanced
241	Kenya/	45802	Advanced
242	Kenya*.cp,in,jw,mp.	110143	Advanced
243	Liberia/	3651	Advanced
244	Liberia*.cp,in,jw,mp.	6151	Advanced
245	Madagascar/	9500	Advanced
246	(Madagasca* or Malagasy Republic).cp,in,jw,mp.	16045	Advanced
247	Malawi/	15425	Advanced
248	(Malawi* or Nyasaland).cp,in,jw,mp.	24406	Advanced
249	Mali/	7677	Advanced
250	Mali*.cp,in,jw,mp.	1529474	Advanced
251	Mauritania/	1375	Advanced
252	Mauritania*.cp,in,jw,mp.	2215	Advanced
253	Mozambique/	7310	Advanced
254	(Mozambi* or Portuguese East Africa).cp,in,jw,mp.	12219	Advanced
255	Niger/	4346	Advanced
256	(Niger not (Aspergillus or Peptococcus or Schizothorax or Cruciferae or Gobius or Lasius or Agelastes or Melanosuchus or radish or Parastromateus or Orius or Apergillus or Parastromateus or Stomoxys)).cp,in,jw,mp.	11622	Advanced
257	Rwanda/	6725	Advanced
258	(Rwanda* or Ruanda*).cp,in,jw,mp.	11039	Advanced
259	Sierra Leone/	4600	Advanced
260	Sierra Leone*.cp,in,jw,mp.	7230	Advanced
261	Somalia/	4197	Advanced
262	Somali*.cp,in,jw,mp.	8619	Advanced
263	Tanzania/	32576	Advanced
264	Tanzania*.cp,in,jw,mp.	48125	Advanced
265	Togo/	3452	Advanced
266	Togo*.cp,in,jw,mp.	8974	Advanced
267	Uganda/	34870	Advanced
268	Uganda*.cp,in,jw,mp.	67334	Advanced
269	Zimbabwe/	15699	Advanced
270	(Zimbabwe* or Rhodesia*).cp,in,jw,mp.	31240	Advanced
271	Cameroon/	16397	Advanced
272	Cameroon*.cp,in,jw,mp.	31218	Advanced
273	Cape Verde/	624	Advanced
274	Cape Verde*.cp,in,jw,mp.	1521	Advanced
275	Congo/	6707	Advanced
276	(congo* not ((democratic republic adj3 congo) or congo red or crimean-congo)).cp,in,jw,mp.	18230	Advanced
277	Cote d’Ivoire/	9588	Advanced
278	(Cote d’Ivoire or Ivory Coast).cp,in,jw,mp.	17382	Advanced
279	Ghana/	23375	Advanced
280	(Ghan* or Gold Coast).cp,in,jw,mp.	80459	Advanced
281	Lesotho/	1422	Advanced
282	(Lesotho or Basutoland).cp,in,jw,mp.	2419	Advanced
283	Nigeria/	86757	Advanced
284	Nigeria*.cp,in,jw,mp.	183806	Advanced
285	Atlantic Islands/	1622	Advanced
286	(sao tome adj2 principe).cp,in,jw,mp.	484	Advanced
287	Senegal/	15789	Advanced
288	Senegal*.cp,in,jw,mp.	36157	Advanced
289	Sudan/	15334	Advanced
290	Sudan*.cp,in,jw,mp.	36837	Advanced
291	Swaziland/	1918	Advanced
292	Swazi*.cp,in,jw,mp.	3736	Advanced
293	Zambia/	13256	Advanced
294	(Zambia* or Northern Rhodesia*).cp,in,jw,mp.	21380	Advanced
295	Angola/	3012	Advanced
296	Angola*.cp,in,jw,mp.	5174	Advanced
297	Botswana/	5298	Advanced
298	(Botswana* or Bechuanaland or Kalahari).cp,in,jw,mp.	9991	Advanced
299	Gabon/	4399	Advanced
300	Gabon*.cp,in,jw,mp.	8593	Advanced
301	Mauritius/	1812	Advanced
302	(Mauriti* or Agalega Islands).cp,in,jw,mp.	6514	Advanced
303	Namibia/	3077	Advanced
304	Namibia*.cp,in,jw,mp.	5578	Advanced
305	Seychelles/	944	Advanced
306	Seychelles.cp,in,jw,mp.	1946	Advanced
307	South Africa/	106477	Advanced
308	South Africa*.cp,in,jw,mp.	359112	Advanced
309	or/29–308	16250440	Advanced
310	or/1–18	9906832	Advanced
311	310 and 28 and 309	15681	Advanced
312	exp animals/ not humans.sh.	32246772	Advanced
313	311 not 312	5415	Advanced

**Table S2.** Characteristics of individual studies.

Breast cancer			
Author, funding, ethics	Study design and setting	Study characteristics	Outcome
**Belicza 2002** **Funding: Not reported** **Ethics: Not reported**	Design: Ecological Conflict: Croatian War of Independence (1991 to 1995) Jurisdiction: City Setting: Hospital Exposure: Uniform	Study year: 1980–2000 Sample size: 2,274 Age: Not reported % Male: 0 Time between exposure and outcome: 15 years NOS Score: 4 Selection: 3 Comparability: 0 Outcome: 1	Outcome: Breast cancer Measured: Hospital records Epidemiological measure: Incidence Effect estimate and direction (recalculated): Pre-conflict: Mean 142.2 cases/year During conflict: Mean 66.4 cases/year Post-conflict: Mean 75.6 cases/year Difference: Pre- versus during conflict: −75.8 (95% CI −128.1 to −23.5) Decrease Pre- versus post-conflict: −66.6 (95% CI −119.4 to −13.8) Decrease During versus post-conflict: 9.2 (95% CI −6.3 to 24.7) No change
**Dmitrovic 2006** **Funding: Yes** **Ethics: Not reported**	Design: Ecological Conflict: Croatian War of Independence (1991 to 1995) Jurisdiction: City Setting: Hospital Exposure: Uniform	Study year: 1990–1993 Sample size: 118 Age: Not reported % Male: Not reported Time between exposure and outcome: 3 years NOS Score: 4 Selection: 3 Comparability: 0 Outcome: 1	Outcome: Malignant breast cancer Measured: Pathohistological confirmation Epidemiological measure: Incidence Effect estimate and direction (recalculated): Pre-conflict: 86 cases in 2 years During conflict: 32 cases in 2 years Difference: −54.0 (95% CI −75.3 to −32.7) Decrease
**Fajdic 2009** **Funding: Not reported** **Ethics: No**	Design: Ecological Conflict: Croatian War of Independence (1991 to 1995) Jurisdiction: Subnational Setting: Hospital Exposure: Uniform	Study year: 1986–2000 Sample size: 514 Age: Not reported % Male: 1 Time between exposure and outcome: 14 years NOS Score: 4 Selection: 3 Comparability: 0 Outcome: 1	Outcome: Breast cancer Measured: Histological confirmation Epidemiological measure: Incidence Effect estimate and direction (recalculated): Pre-conflict: 140 cases in 5 years During conflict: 156 cases in 5 years Post-conflict: 223 cases in 5 years Difference: Pre- versus during conflict: 16.0 (95% CI −18.2 to 49.2) No change Pre- versus post-conflict: 83.0 (95% CI 44.3 to 120.7) Increase During versus post-conflict: 67.0 (95% CI 28.8 to 105.2) Increase
**Karelovic 2002** **Funding: Not reported** **Ethics: Not reported**	Design: Ecological Conflict: Croatian War of Independence (1991 to 1995) Jurisdiction: City Setting: Hospital Exposure: Uniform	Study year: 1988–1993 Sample size: 768 Age: 19 to 88 years % Male: 2 Time between exposure and outcome: 7 years NOS Score: 4 Selection: 3 Comparability: 0 Outcome: 1	Outcome: Breast cancer Measured: Not reported Epidemiological measure: Incidence Effect estimate and direction (recalculated): Pre-conflict: Mean 129 cases/year During conflict: Mean 127 cases/year Difference: −2.7 (95% CI −29.2 to 23.9) No change”
**Korda-Vidic 2015** **Funding: Not reported** **Ethics: Yes**	Design: Case control Conflict: Bosnian War (1992–1995) Jurisdiction: National Setting: Hospital Exposure: Exposed to specific armed conflict events	Study year: 2008–2009 Sample size: 200 Age: 58 years % Male: 0 Time between exposure and outcome: 17 years NOS Score: 6 Selection: 3 Comparability: 2 Outcome: 1	Outcome: Breast cancer Measured: Hospital records Epidemiological measure: Odds ratio Effect estimate and direction (recalculated): 1.55 (95% CI 1.37 to 1.73) Increase
**Koupil 2009** **Funding: Yes** **Ethics: Yes**	Design: Cohort Conflict: Siege of Leningrad (1941–1944) Jurisdiction: City Setting: Community Exposure: Time of birth	Study year: 2005 Sample size: 4,172 Age: 49 years % Male: 78 Time between exposure and outcome: 64 years NOS Score: 7 Selection: 3 Comparability: 2 Outcome: 2	Outcome: Breast cancer mortality Measured: Death certificates coded by physicians (ICD-8) Epidemiological measure: Adjusted hazard ratios Effect estimate and direction (as reported): 2.40 (95% CI 0.86 to 6.72) No change
**Petrovic 2003** **Funding: Not reported** **Ethics: Not reported**	Design: Ecological Conflict: NATO bombing of Yugoslavia (1999) Jurisdiction: City Setting: Hospital Exposure: Uniform	Study year: 1986–1999 Sample size: 1,206 Age: Not reported % Male: 0 Time between exposure and outcome: 13 years NOS Score: 5 Selection: 4 Comparability: 0 Outcome: 1”	Outcome: Breast cancer Measured: Hospital records Epidemiological measure: Incidence Effect estimate and direction (recalculated): Pre-conflict: Mean 67.2/year During conflict: Mean 80.2/year Difference: 13.0 (95% CI 4.1 to 21.9) Increase
**Vagero 2013** **Funding: Yes** **Ethics: Yes**	Design: Cohort Conflict: Siege of Leningrad (1941–1944) Jurisdiction: City Setting: Community Exposure: Time of birth	Study year: 1975–1977 (men); 1980–1982 (women) Sample size: 5,327 Age: Not reported % Male: 73 Time between exposure and outcome: 41 years NOS Score: 3 Selection: 2 Comparability: 0 Outcome: 1	Outcome: Breast cancer mortality Measured: Death certificates coded by physicians (ICD-8) Epidemiological measure: Relative risk Effect estimate and direction (as reported): 1.89 (95% CI 0.83 to 4.31) No change

Cervical cancer			
Author, funding, ethics	Study design and setting	Study characteristics	Outcome
**Huynh 2004** **Funding: Not reported** **Ethics: Yes**	Design: Case control Conflict: Vietnam War (1955–1975) Jurisdiction: City Setting: Hospital Exposure: Husband in army	Study year: 1996 Sample size: 225 Age: Not reported % Male: 0 Time between exposure and outcome: 21 years NOS Score: 5 Selection: 2 Comparability: 2 Outcome: 1	Outcome: Cervical cancer Measured: Biopsy confirmed Epidemiological measure: Odds ratio Effect estimate and direction (as reported): 1.32 (95% CI 1.00 to 1.75) Increase
**Milojkovic 2005** **Funding: Not reported** **Ethics: Not reported**	Design: Ecological Conflict: Croatian War of Independence (1991 to 1995) Jurisdiction: City Setting: Hospital Exposure: Uniform	Study year: 1984−2002 Sample size: 567 Age: Not reported % Male: Not reported Time between exposure and outcome: 10 years NOS Score: 4 Selection: 3 Comparability: 0 Outcome: 1	Outcome: Cervical cancer Measured: Histological confirmation Epidemiological measure: Incidence Effect estimate and direction (recalculated): Pre-conflict: 214 cases in 6 years During conflict: 142 cases in 6 years Post-conflict: 211 cases in 6 years Difference: Pre- versus during conflict: −72.0 (95% CI −109.0 to −35.0) Decrease Pre- versus post-conflict: −3.0 (95% CI −39.8 to 33.8) No change During versus post-conflict: 69.0 (95% CI 32.2 to 105.8) Increase
**Papathanasiou 2005** **Funding: Not reported** **Ethics: Not reported**	Design: Ecological Conflict: NATO bombing of Yugoslavia (1999) Jurisdiction: Subnational Setting: Hospital Exposure: Uniform	Study year: 1997–2002 Sample size: 742 Age: 37–40 years %Male: 0 Time between exposure and outcome: 3 years NOS Score: 4 Selection: 3 Comparability: 0 Outcome: 1	Outcome: Cervical cancer Measured: Hospital records Epidemiological measure: Odds ratio Effect estimate and direction (recalculated): Exposed: 3/5,485 smears Unexposed: 9 cases/30,007 smears OR: 1.82 (95% CI 0.52 to 3.13) No change
**Papathanasiou 2005** **Funding: Not reported** **Ethics: Not reported**	Design: Ecological Conflict: NATO bombing of Yugoslavia (1999) Jurisdiction: Subnational Setting: Hospital Exposure: Uniform	Study year: 1997–2002 Sample size: 742 Age: 37–40 years %Male: 0 Time between exposure and outcome: 3 years NOS Score: 4 Selection: 3 Comparability: 0 Outcome: 1	Outcome: CIN 1–3 Measured: Hospital records Epidemiological measure: Incidence Effect estimate and direction (recalculated): Exposed: 61 cases/5,485 smears Unexposed: 266 cases/30,007 smears AOR 1.26 (95% CI 0.98 to 1.54) No change

Cancers of the central nervous system			
Author, funding, ethics	Study design and setting	Study characteristics	Outcome
**Alajbegovic 2002** **Funding: Not reported** **Ethics: Not reported**	Design: Ecological Conflict: Bosnian War (1992–1995) Jurisdiction: City Setting: Hospital Exposure: Uniform	Study year: 1990–1999 Sample size: 279 Age: 60 years %Male: 58 Time between exposure and outcome: 7 years NOS Score: 4 Selection: 3 Comparability: 0 Outcome: 1	Outcome: CNS Cancers Measured: Hospital records Epidemiological measure: Incidence Effect estimate and direction (recalculated): Pre-conflict: 39.4 cases/year During conflict: 18.1 cases/year Post-conflict: 30.8 cases/year Difference: Pre- versus during conflict: −21.3 (95% CI −36.2 to −6.4) Decrease Pre- versus post-conflict: −8.6 (95% CI −22.3 to 5.1) No change During versus post-conflict: 12.7 (95% CI −1.0 to 26.4) No change
**Telarovic 2006** **Funding: Not reported** **Ethics: Not reported**	Design: Ecological Conflict: Croatian War of Independence (1991 to 1995) Jurisdiction: Subnational Setting: Hospital Exposure: Uniform	Study year: 1986–2000 Sample size: 364 Age: 57 years % Male: 58 Time between exposure and outcome: 9 years NOS Score: 4 Selection: 3 Comparability: 0 Outcome: 1	Outcome: Intracranial tumours Measured: CT, EEG, NMR, histological evaluation Epidemiology: Incidence Effect direction: Pre- versus during conflict: Increase Pre- versus post-conflict: Increase During- versus post-conflict: No change

Colon cancer			
Author, funding, ethics	Study design and setting	Study characteristics	Outcome
**Dmitrovic 2006** **Funding: Yes** **Ethics: Not reported**	Design: Ecological Conflict: Croatian War of Independence (1991 to 1995) Jurisdiction: City Setting: Hospital Exposure: Uniform	Study year: 1990–1993 Sample size: 98 Age: Not reported % Male: Not reported Time between exposure and outcome: 3 years NOS Score: 4 Selection: 3 Comparability: 0 Outcome: 1	Outcome: Malignant colon cancer Measured: Pathohistological confirmation Epidemiological measure: Incidence Effect estimate and direction (recalculated): Pre-conflict: 61 cases in 2 years During conflict: 37 cases in 2 years Difference: −24.0 (95% CI −43.4 to −4.6) Decrease
**Koupil 2009** **Funding: Yes** **Ethics: Yes**	Design: Cohort Conflict: Siege of Leningrad (1941–1944) Jurisdiction: City Setting: Community Exposure: Time of birth	Study year: 2005 Sample size: 4,172 Age: 49 years % Male: 78 Time between exposure and outcome: 64 years NOS Score: 7 Selection: 3 Comparability: 2 Outcome: 2	Outcome: Colorectal cancer mortality Measured: Death certificates coded by physicians (ICD-8) Epidemiological measure: Adjusted hazard ratios Effect estimate and direction (as reported): 0.81 (95% CI 0.50 to 1.33) No change

Cancer of the corpus			
Author, funding, ethics	Study design and setting	Study characteristics	Outcome
**Milojkovic 2005** **Funding: Not reported** **Ethics: Not reported**	Design: Ecological Conflict: Croatian War of Independence (1991 to 1995) Jurisdiction: City Setting: Hospital Exposure: Uniform	Study year: 1984–2002 Sample size: 451 Age: Not reported % Male: Not reported Time between exposure and outcome: 10 years NOS Score: 4 Selection: 3 Comparability: 0 Outcome: 1	Outcome: Corpus cancer, unspecified Measured: Histological confirmation Epidemiological measure: Incidence Effect estimate and direction (recalculated): Pre-conflict: 133 cases in 6 years During conflict: 126 cases in 6 years Post-conflict: 192 cases in 6 years Difference: Pre- versus during conflict: −7.0 (95% CI −38.5 to 24.5) No change Pre- versus post-conflict: 59.0 (95% CI 24.0 to 94.0) Increase During versus post-conflict: 66.0 (95% CI 31.0 to 101.0) Increase

Haematological cancers			
Author, funding, ethics	Study design and setting	Study characteristics	Outcome
**Labar 2004** **Funding: Not reported** **Ethics: Not reported**	Design: Ecological Conflict: Croatian War of Independence (1991 to 1995) Jurisdiction: National Setting: Community Exposure: Uniform	Study year: 1986−1998 Sample size: 580 Age: 0–14 years %Male: Not reported Time between exposure and outcome: 7 years NOS Score: 5 Selection: 3 Comparability: 1 Outcome: 1	Outcome: Haematological malignancies Measured: Cancer Registry of Croatia Epidemiological measure: Incidence Effect estimate and direction (recalculated): Pre-conflict: 249 cases in 5 years During conflict: 216 cases in 5 years Post-conflict: 115 cases in 5 years Difference: Pre- versus during conflict: −33.0 (95% CI −75.3 to 9.3) No change Pre- versus post-conflict: −134.0 (95% CI −169.7 to −98.3) Decrease During versus post-conflict: −101.0 (95% CI −136.7 to −65.3) Decrease
			Outcome: Hodgkin’s lymphoma Measured: Cancer Registry of Croatia Epidemiological measure: Incidence Effect estimate and direction (recalculated): Pre-conflict: 29 cases in 5 years During conflict: 25 cases in 5 years Post-conflict: 10 cases in 5 years Difference: Pre- versus during conflict: −4.0 (95% CI −18.4 to 10.4) No change Pre- versus post-conflict: −19.0 (95% CI −30.6 to −15.0) Decrease During versus post-conflict: −15.0 (95% CI −26.6 to −3.4) Decrease Outcome: Non-Hodgkin’s lymphoma Measured: Cancer Registry of Croatia Epidemiological measure: Incidence Effect estimate and direction (recalculated): Pre-conflict: 54 cases in 5 years During conflict: 44 cases in 5 years Post-conflict: 18 cases in 5 years Difference: Pre- versus during conflict: −10.0 (95% CI −29.4 to 9.4) No change Pre- versus post-conflict: −36.0 (95% CI −51.4 to −20.6) Decrease During versus post-conflict: −26.0 (95% CI −41.4 to −10.6) Decrease Outcome: Lymphatic leukaemia Measured: Cancer Registry of Croatia Epidemiological measure: Incidence Effect estimate and direction (recalculated): Pre-conflict: 129 cases in 5 years During conflict: 132 cases in 5 years Post-conflict: 69 cases in 5 years
			Difference: Pre- versus during conflict: 3.0 (95% CI −28.7 to 34.7) No change Pre- versus post-conflict: −61.0 (95% CI −88.7 to −33.3) Decrease During versus post-conflict: −64.0 (95% CI −91.7 to −36.3) Decrease Outcome: Myeloid leukaemia Measured: Cancer Registry of Croatia Epidemiological measure: Incidence Effect estimate and direction (recalculated): Pre-conflict: 37 cases in 5 years During conflict: 15 cases in 5 years Post-conflict: 19 cases in 5 years Difference: Pre- versus during conflict: −22.0 (95% CI −36.1 to −7.9) Decrease Pre- versus post-conflict: −18.0 (95% CI −29.4 to −6.6) Decrease During versus post-conflict: 4.0 (95% CI −7.4 to 15.4) No change
**Labar 2004** **Funding: Not reported** **Ethics: Not reported**	Design: Ecological Conflict: Croatian War of Independence (1991 to 1995) Jurisdiction: National Setting: Community Exposure: Chemical damage	Study year: 1986–1998 Sample size: 580 Age: 0–14 years %Male: Not reported Time between exposure and outcome: 7 years NOS Score: 5 Selection: 3 Comparability: 1 Outcome: 1	Outcome: Hodgkin’s lymphoma Measured: Cancer Registry of Croatia Epidemiological measure: Incidence Effect estimate and direction (recalculated): Pre-conflict: 3 cases in 5 years During conflict: 3 cases in 5 years Post-conflict: 0 cases in 5 years Difference: Pre- versus during conflict: 0.0 (95% CI −4.8 to 4.8) No change Pre- versus post-conflict: −3.0 (95% CI −6.4 to 0.4) No change During versus post-conflict: −3.0 (95% CI −6.4 to 0.4) No change
			Outcome: Non-Hodgkin’s lymphoma Measured: Cancer Registry of Croatia Epidemiological measure: Incidence Effect estimate and direction (recalculated): Pre-conflict: 1 case in 5 years During conflict: 3 cases in 5 years Post-conflict: 2 cases in 5 years Difference: Pre- versus during conflict: 2.0 (95% CI −1.9 to 5.9) No change Pre- versus post-conflict: (95% CI −3.4 to 5.4) No change During versus post-conflict: −1.0 (95% CI −5.4 to 3.4) No change Outcome: Lymphatic leukaemia Measured: Cancer Registry of Croatia Epidemiological measure: Incidence Effect estimate and direction (recalculated): Pre-conflict: 12 cases in 5 years During conflict: 6 cases in 5 years Post-conflict: 2 cases in 5 years Difference: Pre- versus during conflict: −6.0 (95% CI −14.3 to 2.3) No change Pre- versus post-conflict: −10.0 (95% CI −15.5 to 4.5) Decrease During versus post-conflict: −4.0 (95% CI −9.5 to 1.5) No change Outcome: Myeloid leukaemia Measured: Cancer Registry of Croatia Epidemiological measure: Incidence Effect estimate and direction (recalculated): Pre-conflict: 3 cases in 5 years During conflict: 1 case in 5 years Post-conflict: 0 cases in 5 years
			Difference: Pre- versus during conflict: −2.0 (95% CI −5.9 to 1.9) No change Pre- versus post-conflict: −3.0 (95% CI −5.0 to −1.0) No change During versus post-conflict: −1.0 (95% CI −3.0 to 1.0) No change
**Labar 2004** **Funding: Not reported** **Ethics: Not reported**	Design: Ecological Conflict: Croatian War of Independence (1991 to 1995) Jurisdiction: National Setting: Community Exposure: Chemical damage	Study year: 1986–1998 Sample size: 580 Age: 0–14 years %Male: Not reported Time between exposure and outcome: 7 years NOS Score: 5 Selection: 3 Comparability: 1 Outcome: 1	Outcome: Hodgkin’s lymphoma Measured: Cancer Registry of Croatia Epidemiological measure: Incidence Effect estimate and direction (recalculated): Pre-conflict: 3 cases in 5 years During conflict: 3 cases in 5 years Post-conflict: 0 cases in 5 years Difference: Pre- versus during conflict: 0.0 (95% CI −4.8 to 4.8) No change Pre- versus post-conflict: −3.0 (95% CI −6.4 to 0.4) No change During versus post-conflict: −3.0 (95% CI −6.4 to 0.4) No change Outcome: Non-Hodgkin’s lymphoma Measured: Cancer Registry of Croatia Epidemiological measure: Incidence Effect estimate and direction (recalculated): Pre-conflict: 1 case in 5 years During conflict: 3 cases in 5 years Post-conflict: 2 cases in 5 years Difference: Pre- versus during conflict: 2.0 (95% CI −1.9 to 5.9) No change Pre- versus post-conflict: (95% CI −3.4 to 5.4) No change During versus post-conflict: −1.0 (95% CI −5.4 to 3.4) No change
			Outcome: Lymphatic leukaemia Measured: Cancer Registry of Croatia Epidemiological measure: Incidence Effect estimate and direction (recalculated): Pre-conflict: 12 cases in 5 years During conflict: 6 cases in 5 years Post-conflict: 2 cases in 5 years Difference: Pre- versus during conflict: −6.0 (95% CI −14.3 to 2.3) No change Pre- versus post-conflict: −10.0 (95% CI −15.5 to 4.5) Decrease During versus post-conflict: −4.0 (95% CI −9.5 to 1.5) No change Outcome: Myeloid leukaemia Measured: Cancer Registry of Croatia Epidemiological measure: Incidence Effect estimate and direction (recalculated): Pre-conflict: 3 cases in 5 years During conflict: 1 case in 5 years Post-conflict: 0 cases in 5 years Difference: Pre- versus during conflict: −2.0 (95% CI −5.9 to 1.9) No change Pre- versus post-conflict: −3.0 (95% CI −5.0 to −1.0) No change During versus post-conflict: −1.0 (95% CI −3.0 to 1.0) No change
**Labar 2004** **Funding: Not reported** **Ethics: Not reported**	Design: Ecological Conflict: Croatian War of Independence (1991 to 1995) Jurisdiction: National Setting: Community Exposure: Depleted uranium	Study year: 1986−1998 Sample size: 580 Age: 0–14 years %Male: Not reported Time between exposure and outcome: 7 years NOS Score: 5 Selection: 3 Comparability: 1 Outcome: 1	Outcome: Hodgkin’s lymphoma Measured: Cancer Registry of Croatia Epidemiological measure: Incidence Effect estimate and direction (recalculated): Pre-conflict: 8 cases in 5 years During conflict: 9 cases in 5 years Post-conflict: 2 cases in 5 years
			Difference: Pre- versus during conflict: (95% CI −7.1 to 9.1) No change Pre- versus post-conflict: −6.0 (95% CI −12.5 to 0.5) No change During versus post-conflict: −7.0 (95% CI −13.5 to −0.5) Decrease Outcome: Non-Hodgkin’s lymphoma Measured: Cancer Registry of Croatia Epidemiological measure: Incidence Effect estimate and direction (recalculated): Pre-conflict: 14 cases in 5 years During conflict: 12 cases in 5 years Post-conflict: 7 cases in 5 years Difference: Pre- versus during conflict: −2.0 (95% CI −12.0 to 8.0) No change Pre- versus post-conflict: −7.0 (95% CI −15.5 to 1.5) No change During versus post-conflict: −5.0 (95% CI −13.5 to 3.5) No change Outcome: Lymphatic leukaemia Measured: Cancer Registry of Croatia Epidemiological measure: Incidence Effect estimate and direction (recalculated): Pre-conflict: 59 cases in 5 years During conflict: 36 cases in 5 years Post-conflict: 25 cases in 5 years Difference: Pre- versus during conflict: −23.0 (95% CI −42.1 to −3.9) Decrease Pre- versus post-conflict: −34.0 (95% CI −49.3 to −18.7) Decrease During versus post-conflict: −11.0 (95% CI −26.3 to 4.3) No change
			Outcome: Myeloid leukaemia Measured: Cancer Registry of Croatia Epidemiological measure: Incidence Effect estimate and direction (recalculated): Pre-conflict: 13 cases in 5 years During conflict: 3 cases in 5 years Post-conflict: 5 cases in 5 years Difference: Pre- versus during conflict: −10.0 (95% CI −17.8 to −2.2) Decrease Pre- versus post-conflict: −8.0 (95% CI −13.5 to −2.5) No change During versus post-conflict: 2.0 (95% CI −3.5 to 7.5) No change
**Labar 2004** **Funding: Not reported** **Ethics: Not reported**	Design: Ecological Conflict: Croatian War of Independence (1991 to 1995) Jurisdiction: National Setting: Community Exposure: Population mixing	Study year: 1986–1998 Sample size: 580 Age: 0–14 years %Male: Not reported Time between exposure and outcome: 7 years NOS Score: 5 Selection: 3 Comparability: 1 Outcome: 1	Outcome: Hodgkin’s lymphoma Measured: Cancer Registry of Croatia Epidemiological measure: Incidence Effect estimate and direction (recalculated): Pre-conflict: 15 cases in 5 years During conflict: 4 cases in 5 years Post-conflict: 1 case in 5 years Difference: Pre- versus during conflict: −11.0 (95% CI −19.5 to −2.5) Decrease Pre- versus post-conflict: −14.0 (95% CI −18.4 to −9.6) Decrease During versus post-conflict: −3.0 (95% CI −7.4 to 1.4) No change Outcome: Non-Hodgkin’s lymphoma Measured: Cancer Registry of Croatia Epidemiological measure: Incidence Effect estimate and direction (recalculated): Pre-conflict: 22 cases in 5 years During conflict: 16 cases in 5 years Post-conflict: 8 cases in 5 years
			Difference: Pre- versus during conflict: −6.0 (95% CI −18.1 to 6.1) No change Pre- versus post-conflict: −14.0 (95% CI −23.6 to −4.4) Decrease During versus post-conflict: −8.0 (95% CI −17.6 to 1.6) No change Outcome: Lymphatic leukaemia Measured: Cancer Registry of Croatia Epidemiological measure: Incidence Effect estimate and direction (recalculated): Pre-conflict: 41 cases in 5 years During conflict: 59 cases in 5 years Post-conflict: 26 cases in 5 years Difference: Pre- versus during conflict: 18.0 (95% CI −1.6 to 37.6) No change Pre- versus post-conflict: −15.0 (95% CI −33.1 to 3.1) No change During versus post-conflict: −33.0 (95% CI −51.1 to −14.9) Decrease Outcome: Myeloid leukaemia Measured: Cancer Registry of Croatia Epidemiological measure: Incidence Effect estimate and direction (recalculated): Pre-conflict: 12 cases in 5 years During conflict: 5 cases in 5 years Post-conflict: 7 cases in 5 years Difference: Pre- versus during conflict: −7.0 (95% CI −15.1 to 1.1) No change Pre- versus post-conflict: −5.0 (95% CI −11.8 to 1.8) No change During versus post-conflict: 2.0 (95% CI −4.8 to 8.8) No change

Lung cancer			
Author, funding, ethics	Study design and setting	Study characteristics	Outcome
**Dmitrovic 2006** Funding: Yes **Ethics: Not reported**	Design: Ecological Conflict: Croatian War of Independence (1991 to 1995) Jurisdiction: City Setting: Hospital Exposure: Uniform	Study year: 1990–1993 Sample size: 121 Age: Not reported % Male: Not reported Time between exposure and outcome: 3 years NOS Score: 4 Selection: 3 Comparability: 0 Outcome: 1	Outcome: Malignant lung cancer Measured: Pathohistological confirmation Epidemiological measure: Incidence Effect estimate and direction (recalculated): Pre-conflict: 63 cases in 2 years During conflict: 58 cases in 2 years Difference: −5.0 (95% CI −26.6 to 16.6) No change
**Koupil 2009** **Funding: Yes** **Ethics: Yes**	Design: Cohort Conflict: Siege of Leningrad (1941–1944) Jurisdiction: City Setting: Community Exposure: Time of birth	Study year: 2005 Sample size: 4,172 Age: 49 years % Male: 78 Time between exposure and outcome: 64 years NOS Score: 7 Selection: 3 Comparability: 2 Outcome: 2	Outcome: Respiratory cancer mortality Measured: Death certificates coded by physicians (ICD-8) Epidemiological measure: Adjusted hazard ratios Effect estimate and direction (as reported): 1.29 (95% CI 0.98 to 1.70) No change
**Vagero 2013** **Funding: Yes** **Ethics: Yes**	Design: Cohort Conflict: Siege of Leningrad (1941–1944) Jurisdiction: City Setting: Community Exposure: Time of birth	Study year: 1975–1977 (men); 1980–1982 (women) Sample size: 5,327 Age: Not reported % Male: 73 Time between exposure and outcome: 41 years NOS Score: 3 Selection: 2 Comparability: 0 Outcome: 1	Outcome: Respiratory cancer mortality Measured: Death certificates coded by physicians (ICD-8) Epidemiological measure: Relative risk Effect estimate and direction (as reported): 1.89 (95% CI 0.83 to 4.31) No change

Oropharyngeal cancer			
Author, funding, ethics	Study design and setting	Study characteristics	Outcome
**Ariyawardana 2011** **Funding: Yes** **Ethics: Not reported**	Design: Ecological Conflict: Sri Lankan Civil War (1983–2009) Jurisdiction: National Setting: Hospital Exposure: Uniform	Study year: 1985–2005 Sample size: 6,391 Age: All ages %Male: 75 Time between exposure and outcome: 20 years NOS Score: 5 Selection: 3 Comparability: 1 Outcome: 1	Outcome: Oropharyngeal cancers Measured: Hospital registries Epidemiological measure: Beta coefficient (age standardised) Effect estimate and direction (as reported): - 0.0092, p = 0.043 - Increase Outcome: Lip and oral cavity cancers Measured: Hospital registries Epidemiological measure: Beta coefficient (age standardised) Effect estimate and direction (as reported): −0.17, p = 0.0028 Decrease

Ovarian cancer			
Author, funding, ethics	Study design and setting	Study characteristics	Outcome
**Dmitrovic 2006** **Funding: Yes** **Ethics: Not reported**	Design: Ecological Conflict: Croatian War of Independence (1991 to 1995) Jurisdiction: City Setting: Hospital Exposure: Uniform	Study year: 1990–1993 Sample size: 62 Age: Not reported % Male: Not reported Time between exposure and outcome: 3 years NOS Score: 4 Selection: 3 Comparability: 0 Outcome: 1	Outcome: Malignant ovarian cancer Measured: Pathohistological confirmation Epidemiological measure: Incidence Effect estimate and direction (recalculated): Pre-conflict: 16 cases in 2 years During conflict: 46 cases in 2 years Difference: 30.0 (95% CI 14.6 to 45.4) Increase
**Milojkovic 2005** **Funding: Not reported** **Ethics: Not reported**	Design: Ecological Conflict: Croatian War of Independence (1991 to 1995) Jurisdiction: City Setting: Hospital Exposure: Uniform	Study year: 1984–2002 Sample size: 262 Age: Not reported % Male: Not reported Time between exposure and outcome: 10 years NOS Score: 4 Selection: 3 Comparability: 0 Outcome: 1	Outcome: Ovarian cancer, unspecified Measured: Histological confirmation Epidemiological measure: Incidence Effect estimate and direction (recalculated): Pre-conflict: 90 cases in 6 years During conflict: 85 cases in 6 years Post-conflict: 144 cases in 6 years Difference: Pre- versus during conflict: −5.0 (95% CI −30.9 to 20.9) No change Pre- versus post-conflict: 54.0 (95% CI 24.3 to 83.7) Increase During versus post-conflict: 59.0 (95% CI 29.3 to 88.7) Increase

Pancreatic cancer			
Author, funding, ethics	Study design and setting	Study characteristics	Outcome
**Dmitrovic 2006** **Funding: Yes** **Ethics: Not reported**	Design: Ecological Conflict: Croatian War of Independence (1991 to 1995) Jurisdiction: City Setting: Hospital Exposure: Uniform	Study year: 1990–1993 Sample size: 8 Age: Not reported % Male: Not reported Time between exposure and outcome: 3 years NOS Score: 4 Selection: 3 Comparability: 0 Outcome: 1	Outcome: Malignant pancreatic cancer Measured: Pathohistological confirmation Epidemiological measure: Incidence Effect estimate and direction (recalculated): Pre-conflict: 5 cases in 2 years During conflict: 3 cases in 2 years Difference: −2.0 (95% CI −7.5 to 3.5) No change

Prostate cancer			
Author, funding, ethics	Study design and setting	Study characteristics	Outcome
**Koupil 2009** **Funding: Yes** **Ethics: Yes**	Design: Cohort Conflict: Siege of Leningrad (1941–1944) Jurisdiction: City Setting: Community Exposure: Time of birth	Study year: 2005 Sample size: 4,172 Age: 49 years % Male: 78 Time between exposure and outcome: 64 years NOS Score: 7 Selection: 3 Comparability: 2 Outcome: 2	Outcome: Prostate cancer mortality Measured: Death certificates coded by physicians (ICD-8) Epidemiological measure: Adjusted hazard ratios Effect estimate and direction (as reported): 1.43 (95% CI 0.70 to 2.92) No change

Stomach cancer			
Author, funding, ethics	Study design and setting	Study characteristics	Outcome
**Dmitrovic 2006** **Funding: Yes** **Ethics: Not reported**	Design: Ecological Conflict: Croatian War of Independence (1991 to 1995) Jurisdiction: City Setting: Hospital Exposure: Uniform	Study year: 1990–1993 Sample size: 76 Age: Not reported % Male: Not reported Time between exposure and outcome: 3 years NOS Score: 4 Selection: 3 Comparability: 0 Outcome: 1	Outcome: Malignant stomach cancer Measured: Pathohistological confirmation Epidemiological measure: Incidence Effect estimate and direction (recalculated): Pre-conflict: 17 cases in 2 years During conflict: 59 cases in 2 years Difference: 42.0 (95% CI 24.9 to 59.1) Increase
**Koupil 2009** **Funding: Yes** **Ethics: Yes**	Design: Cohort Conflict: Siege of Leningrad (1941–1944) Jurisdiction: City Setting: Community Exposure: Time of birth	Study year: 2005 Sample size: 4,172 Age: 49 years % Male: 78 Time between exposure and outcome: 64 years NOS Score: 7 Selection: 3 Comparability: 2 Outcome: 2	Outcome: Stomach cancer mortality Measured: Death certificates coded by physicians (ICD-8) Epidemiological measure: Adjusted hazard ratios Effect estimate and direction (as reported): 0.95 (95% CI 0.65–1.37) No change

Testicular cancer			
Author, funding, ethics	Study design and setting	Study characteristics	Outcome
**Dmitrovic 2006** **Funding: Yes** **Ethics: Not reported**	Design: Ecological Conflict: Croatian War of Independence (1991 to 1995) Jurisdiction: City Setting: Hospital Exposure: Uniform	Study year: 1990–1993 Sample size: 26 Age: Not reported % Male: Not reported Time between exposure and outcome: 3 years NOS Score: 4 Selection: 3 Comparability: 0 Outcome: 1	Outcome: Malignant cancer of the testis Measured: Pathohistological confirmation Epidemiological measure: Incidence Effect estimate and direction (recalculated): Pre-conflict: 6 cases in 2 years During conflict: 20 cases in 2 years Difference: 14.0 (95% CI 4.0 to 24.0) Increase

Cancer, unspecified site			
Author, funding, ethics	Study design and setting	Study characteristics	Outcome
**Adib 1998** **Funding: Yes** **Ethics: Not reported**	Design: Ecological Conflict: Lebanese Civil War (1975–1991) Jurisdiction: City Setting: Hospital Exposure: Uniform	Study year: 1983–1994 Sample size: 9,364 Age: 49–52 years % Male: 50 Time between exposure and outcome: 11 years NOS Score: 4 Selection: 3 Comparability: 0 Outcome: 1	Outcome: Unspecified Measured: Pathology and cytology Epidemiological measure: Incidence Effect estimate and direction (recalculated): During conflict: Mean 785.8 cases/year Post-conflict: Mean 802.3 cases/year Difference: 16.5 (95% CI −61.6 to 94.6) No change
**Al-Hashimi 2013** **Funding: None** **Ethics: Not reported**	Design: Ecological Conflict: Unspecified conflicts in Iraq Jurisdiction: Subnational Setting: Hospital Exposure: Uniform	Study year: 1980–2010 Sample size: Not reported Age: Not reported % Male: Not reported Time between exposure and outcome: 30 years NOS Score: 4 Selection: 3 Comparability: 0 Outcome: 1	Outcome: Unspecified Measured: Hospital records Epidemiological measure: Incidence rate Effect estimate and direction (as reported): Pre-conflict (1980 to 1989): 7.8 (95% CI 6.9 to 8.7) During conflict (1990 to 1999): 10.5 (95% CI 10.2 to 10.8) During conflict (2000–2010): 10.2 (95% CI 9.7 to 10.7) Increase
**Dmitrovic 2006** **Funding: Yes** **Ethics: Not reported**	Design: Ecological Conflict: Croatian War of Independence (1991 to 1995) Jurisdiction: City Setting: Hospital Exposure: Uniform	Study year: 1990–1993 Sample size: 509 Age: Not reported % Male: Not reported Time between exposure and outcome: 3 years NOS Score: 4 Selection: 3 Comparability: 0 Outcome: 1	Outcome: Malignant tumours, unspecified Measured: Pathohistological confirmation Epidemiological measure: Incidence Effect estimate and direction (recalculated): Pre-conflict: 254 cases in 2 years During conflict: 255 cases in 2 years Difference: 1.0 (95% CI −–−43.2 to 45.2) No change
**Drljevic 2005** **Funding: Not reported** **Ethics: Not reported**	Design: Ecological Conflict: Bosnian War (1992–1995) Jurisdiction: City Setting: Hospital Exposure: Uniform	Study year: 1992–2000 Sample size: 855 Age: Not reported % Male: 0 Time between exposure and outcome: 9 years NOS Score: 4 Selection: 3 Comparability: 0 Outcome: 1	Outcome: Female genital cancers Measured: Hospital records Epidemiological measure: Incidence Effect estimate and direction (as reported): Pre- versus during conflict: No change Pre- versus post-conflict: Increase
**Hagopian 2013** **Funding: None** **Ethics: Yes**	Design: Cross-sectional Conflict: Iraq War (2003–2011) Jurisdiction: National Setting: Community Exposure: Uniform	Study year: 2001–2011 Sample size: 35,835 Age: Not reported % Male: Not reported Time between exposure and outcome: 9 years NOS Score: 5 Selection: 4 Comparability: 0 Outcome: 1	Outcome: Cancer mortality Measured: Self-reported Epidemiological measure: Incidence Effect estimate and direction (recalculated): Pre-conflict: Mean 9.9 cases/year During conflict: Mean 14.8 cases/year Difference: 4.9 (95% CI 0.4 to 9.4) Increase
**Koupil 2009** **Funding: Yes** **Ethics: Yes**	Design: Cohort Conflict: Siege of Leningrad (1941–1944) Jurisdiction: City Setting: Community Exposure: Time of birth	Study year: 2005 Sample size: 4,172 Age: 49 years % Male: 78 Time between exposure and outcome: 64 years NOS Score: 7 Selection: 3 Comparability: 2 Outcome: 2	Outcome: All cancer mortality Measured: Death certificates coded by physicians (ICD-8) Epidemiological measure: Adjusted hazard ratios Effect estimate and direction (as reported): 1.12 (95% CI 0.95 to 1.31) No change
**Vagero 2013** **Funding: Yes** **Ethics: Yes**	Design: Cohort Conflict: Siege of Leningrad (1941–1944) Jurisdiction: City Setting: Community Exposure: Time of birth	Study year: 1975–1977 (men); 1980–1982 (women) Sample size: 5,327 Age: Not reported % Male: 73 Time between exposure and outcome: 41 years NOS Score: 3 Selection: 2 Comparability: 0 Outcome: 1	Outcome: All cancer mortality Measured: Death certificates coded by physicians (ICD-8) Epidemiological measure: Relative risk Effect estimate and direction (as reported): 1.11 (95% CI 0.97 to 1.27) No change
**Vlajinac 2000** **Funding: Yes** **Ethics: Yes**	Design: Ecological Conflict: Non-specific conflicts following the breakup up Yugoslavia Jurisdiction: Subnational Setting: Community Exposure: Uniform	Study year: 1973–1994 Sample size: Not reported Age: All ages %Male: Not reported Time between exposure and outcome: 21 years NOS Score: 4 Selection: 3 Comparability: 0 Outcome: 1	Outcome: Unspecified cancer mortality Measured: Federal Institute of Statistics, Serbia Epidemiological measure: Beta coefficient Effect estimate and direction (as reported): *y* = 428.01 + 21.427x − 167.61 (War), *p* = 0.031 Decrease

**Table 1.** Study characteristics and methodological quality of 20 included studies.

Characteristic		% (*N*)
**Year of publication**	1999 or earlier	5.0 (1)
	2000–2009	70.0 (14)
	2010 or later	25.0 (5)
**Funding source**	Reported	25.0 (5)
	None declared	10.0 (2)
	Not reported	65.0 (13)
**Ethics approval**	Yes	25.0 (5)
	No	10.0 (2)
	Not reported	65.0 (13)
**Study design**	Ecological	75.0 (15)
	Case-control	10.0 (2)
	Cohort	10.0 (2)
	Cross-sectional	5.0 (1)
**Armed conflict**	Croatian War of Independence (1991–1995)	35.0 (7)
	Bosnian War (1992–1995)	15.0 (3)
	Siege of Leningrad (1941–1944)	10.0 (2)
	NATO bombing of Yugoslavia (1999)	10.0 (2)
	Iraq War (2003–2011)	5.0 (1)
	Unspecified conflicts in Iraq	5.0 (1)
	Lebanese Civil War (1975–1991)	5.0 (1)
	Sri Lankan Civil War (1983–2009)	5.0 (1)
	Vietnam War (1955–1975)	5.0 (1)
	Unspecified conflicts following the breakup of Yugoslavia	5.0 (1)
**Level of jurisdiction**	City	55.0 (11)
	Subnational	25.0 (5)
	National	20.0 (4)
**Setting**	Hospital	70.0 (14)
	Community	30.0 (6)
**Armed conflict exposure measurement**	Uniform exposure to all based on time and place	80.0 (16)
	Exposure based on time of birth	10.0 (2)
	Exposure to specific armed conflict events	5.0 (1)
	Exposure based having a relative in the military	5.0 (1)
**Time between conflict and outcome**	Less than 5 years	15.0 (3)
	5.0–9.9 years	25.0 (5)
	10.0–39.9 years	50.0 (10)
	40 years or more	10.0 (2)
**Newcastle-Ottawa Scale**	Low quality (score <5)	65.0 (13)
	Moderate quality (score 5–6)	25.0 (5)
	High quality (score >6)	10.0 (2)
